# Experiences of the coronavirus disease-19 (COVID-19) pandemic from the perspectives of young people: Rapid qualitative study

**DOI:** 10.1016/j.puhip.2021.100162

**Published:** 2021-11

**Authors:** Harriet Fisher, Helen Lambert, Matthew Hickman, Lucy Yardley, Suzanne Audrey

**Affiliations:** a Population Health Sciences, Bristol Medical School, University of Bristol, Bristol, UK; b School of Psychological Science, University of Bristol, Bristol, UK; c School of Psychology, University of Southampton, Southampton, UK

**Keywords:** Young people, COVID-19 pandemic, Adherence, Public health, Qualitative, Vaccination, Schools

## Abstract

**Objectives:**

Young people are considered at lower risk from coronavirus disease-19 (COVID-19). However, measures to limit the population health impact of the current COVID-19 pandemic have caused significant disruptions to their lives. The objective of this study was to explore the experiences of young people predominantly living in the south-west of England during the COVID-19 pandemic.

**Study design:**

Rapid qualitative study.

**Methods:**

Following advertisement on social media, a purposive sample of young people by age and gender who had expressed an interest were invited to participate. In June 2020, 21 young people (12–17 years) took part in 18 semi-structured interviews, either through a digital platform or by telephone. Interviews were recorded digitally and transcribed verbatim. Thematic analysis was undertaken, assisted by NVvivo Software.

**Results:**

Young people felt the greatest impacts of the pandemic have been disruption to how they learned because of school closures and limited face-to-face interaction with their social networks. There was variation in terms of how satisfied young people were with self-directed learning at home, and some anxieties in relation to its effectiveness outside the school environment. Most young people reported maintaining social relationships remotely, but some young people appeared to have little social interaction outside their household. High levels of adherence to social distancing and handwashing were reported, which could lead to a sense of injustice resulting from visibility of other people breaching social distancing guidance. Young people were willing to be vaccinated against COVID-19 if a vaccine became available, with the greatest motivator being to protect others above themselves.

**Conclusions:**

Young people have experienced significant disruption to their education and social networks during the COVID-19 pandemic. During lockdown, high levels of compliance to government public health guidelines to reduce transmission of COVID-19 were reported by young people. If an effective vaccine is developed, a schools-based vaccination programme could be an efficient method to interrupt transmission to more at-risk populations and prevent further disruptions to young people's education.

## Highlights

• Young people reported mixed experiences of self-directed learning at home.
• Judgements against those not adhering to social-distancing guidelines were apparent.
• Motivations to be vaccinated against COVID-19 were driven by high levels of altruism.

## 1 Background

### 1.1 Coronavirus 2019 (COVID-19) pandemic

Infection with the new coronavirus (severe acute respiratory syndrome coronavirus 2, or SARS-CoV-2) causes novel coronavirus disease 2019 (COVID-19). In March 2020, the World Health Organisation declared the COVID-19 outbreak to be a pandemic [1]. By January 2021, over 100 million cases had been confirmed in addition to over 2 million fatalities [2].

### 1.2 Public health control measures

In England, public health control measures to reduce transmission were introduced. In late March 2020, schools were closed nationally, and ‘lockdown’ measures were put in place where the public were allowed to leave their homes only for exercise and for grocery shopping. From mid-May, the general public were allowed outside for unlimited exercise and to meet one other person at a minimum distance of two metres. From mid-June, primary and secondary schools were partially opened for priority year groups, and up to six people were allowed to meet outside.

### 1.3 Potential impact of the pandemic on young people

In England, there is strong accumulating evidence that people aged over 70 years are disproportionately affected by COVID-19 [3]. Young people (defined in the study according to the World Health Organisation criteria as aged between 10 and 24 years [4]) are much less likely to be adversely affected by COVID-19 in relation to their physical health. However, they have had to adjust to dramatic changes in their daily routines, homelife, and education as a result of COVID-19 public health measures.

Concerns have been raised that young people's social and emotional well-being has been negatively affected by these changes. A survey showed a deterioration in symptoms and increased feelings of loneliness and isolation among young people with existing mental health issues [5]. Similarly, a survey undertaken in the north of England reported young people have increased anxiety and worries [6]. Concurrently, young people have recently become subject to increased negative scrutiny by local media outlets [7,8] following reports that they are disproportionately represented in new cases of COVID-19 across Europe [9].

### 1.4 Aims and objectives

The overall aim of this rapid qualitative study is to examine the experiences of the COVID-19 pandemic from the perspectives of young people. The specific objectives are to:

(i) explore the social impact of the COVID-19 pandemic on young people;
(ii) examine the extent to which young people are implementing COVID-19 public health guidance, and;
(iii) consider the acceptability of vaccination against COVID-19 among young people.

## 2 Methods

### 2.1 Study design

A rapid qualitative study.

### 2.2 Recruitment

Recruitment and data collection took place in June 2020. The study was initially promoted by an email and social media accounts associated with University of Bristol research and advisory groups. These posts were then more widely disseminated through recipients’ own social network and social media accounts. Overall, 121 expressions of interest were received from young people or their parents. The age and gender of the potential participants were ascertained. Ethnicity and sociodemographic data were not collected.

### 2.3 Sampling strategy

To ensure the study sample was balanced by gender and age category, a purposive sample of young people were selected to participate. The characteristics of the sample by age and gender are provided (Table 1). Most participants lived in Bristol or the immediate surrounding areas, with three participants based in London and two in South Wales.

**Table 1:** Summary of participants by age and gender.

Gender/Age	12–14	15–17	Total
Male	6	5	**11**
Female	5	5	**10**
**Total**	**11**	**10**	**21**

### 2.4 Data collection

Topic guides were developed to explore young people's experiences during the COVID-19 pandemic. Prior to the interviews taking place, verbal or written parental consent was recorded for participants aged 15 years or below. Verbal or written assent was also obtained from all participants, which included permission to digitally record the interview. Semi-structured interviews took place remotely either by a digital platform or telephone. Depending on their preference, young people were interviewed individually, or with a nominated peer or family member.

### 2.5 Analysis

Interview recordings were transcribed, anonymised, and double checked for accuracy. Thematic analysis [10] was undertaken assisted by NVivo 12 software. Familiarisation with the dataset began by reading and re-reading the transcripts. Sections of text were coded, with multiple codes being allocated where appropriate. Coding was simultaneously inductive (emerging from the data in the transcripts) and deductive (based on the research questions). Similar codes were grouped together to create a thematic framework comprising a hierarchy of themes and sub-themes, within which similarities and differences were explored. Interpretation of the data was discussed at a consensus meeting (HF, SA) as analysis progressed.

## 3 Results

Twenty-one young people participated in 18 interviews (depending on their preference to be interviewed alone or with a peer or family member).

The findings are reported by the following themes and sub-themes related to the objectives of the study: (i) social impact of COVID-19 pandemic on young people (‘schooling and education’, ‘disruption to social networks’, and ‘hobbies and extracurricular activities’); (ii) implementation of COVID-19 government guidelines (‘social distancing’, ‘handwashing and hygiene’, ‘self-isolation’, and ‘enforcement of rules’), and: (iii) acceptability of vaccination against COVID-19. Illustrative quotations were chosen, because they were expressed concisely and typify responses relating to the themes (Table 2, Table 3, Table 4).

**Table 2:** Impact of the COVID-19 pandemic on young people.

Sub-theme	
Schooling and education	‘*We have [software name] and then they set work on there as if it was homework but it's actually just lesson plans. Then we have to do all that work and send it back*.’ [Participant 18, male, 13 years old]
	‘*We're teaching ourselves everything and they would say email us if you have any problems and I'd email a teacher, I emailed about three, and none of them replied, so it was very difficult to get help*.’ [Participant 11, female, 17 years old]
	‘*I think it's been quite good because I think I've learnt a lot more than I would at school*.’ [Participant 6, female, 13 years old]
	‘*I find it a lot easier to learn at school, and probably learn more at school, because I don't read out what you've actually learnt, so you can do the work but I don't read it out or try to remember*.’ [Participant 16, male, 15 years old]
	‘*It's been harder to learn and I feel quite behind and I'm getting a bit stressed about catching up when I go back to school, and I know a lot of my friends are as well*.’ [Participant 2, female, 14 years old]
	‘*I would say it's not that much of a big deal but if you're like Year Ten, you're doing the GCSEs or A-levels then I think it might be more of a problem*.’ [Participant 7, male, 13 years old]
	‘*At first it really stressed me out because I was thinking I wasn't going to get the grades that I deserved … but I think looking back now I did miss out on a lot of stress*.’ [Participant 10, female, 16 years old]
	‘*If you're mates with your teacher, they might just subconsciously give you a better mark because they like you. It could just be the complete opposite. Lucky enough for me though, I was friends with all my teachers*.’ [Participant 20, male, 16 years old]
	‘*I think it's the middle of July that we're going to be going back for two weeks for 2 h per subject, so if you're doing three subjects that's 6 h [laughs]. It seems a bit useless to me*.’ [Participant 11, female, 17 years old]
	‘*I'm not sure how long they're planning to keep people distanced and stuff like that … hopefully we'll have very small classes*.’ [Participant 13, female, 15 years old]
	‘*I don't think I would be that worried because I know the teachers are going to be quite strict and they've said if you don't distance or if you don't listen to what the teacher is saying, they'll say you can't come back into school*.’ [Participant 2, female, 14 years old]
	‘*After a bit you do miss your friends and stuff. It'd be quite nice to go back to school*.’ [Participant 12, male, 13 years old]
	‘*I would probably prefer it if we didn't have to go to school ‘*cos *I'd prefer to stay at home than go to school anyway*.’ [Participant 8, male, 13 years old]
	‘*I felt kind of relieved [that the school closed].*
	*That's a small weight off my shoulders ‘*cos *I don't have to get up in the morning*.’ [Participant 7, male, 13 years old]
Disruption to social networks	‘*I thought it was going to be really horrible and everything, but you talk to people so much on your phone, constantly, just conversations or FaceTime*.’ [Participant 15, female, 15 years old]
	‘*I think I've been in my room for most of it, except for mealtimes*.’ [Participant 4, male, 12 years old]
	‘*I'm not really a sociable person so I don't really meet up with friends at all*.’ [Participant 19, male, 15 years old]
	‘*We'd take turns meeting one-to-one and now all of a sudden, we can meet in six which is really nice, so we take advantage of that, maybe once or twice a week. Just sit on the [park] in a socially distanced circle having a barbeque*.’ [Participant 14, male, 17 years old]
	‘*Normally you see your friends every day without having to organise it and arrange it and check with parents. But at the moment you have to plan a date, and everything has to be a bit more structured. It's not like you just see them every day*.’ [Participant 3, female, 12 years old]
Hobbies and extracurricular activities	‘*I have Italian friends, and I tried to make pizza Face Timing them. At the beginning it was bad, it was very bad, and right now it's so good!*’ [Participant 17, male, 16 years old]
	‘*Me and my mum just did so many projects. We made masks for all the relatives and we've got a sewing machine, so I was sewing some clothes*.’ [Participant 10, female, 16 years old]
	‘*I have a drama school, they're doing that on-line now. Every week they've been setting us tasks, make videos for them and putting it on to one big compilation … It's fun because they give us something to do instead of just sitting around playing games*.’ [Participant 20, male, 16 years old]
	‘*We'd go with our mum and dad usually and now we can go [bike riding] by ourselves*.’ [Participant 1, female, 12 years old]

**Table 3:** Implementation of COVID-19 government guidelines.

Sub-theme	
Social distancing	‘*Me and my family has followed all the rules and stayed at home for as long as we can and 2 m apart and when we have to go shopping we won't touch certain items we don't have to*.’ [Participant 8, male, 13 years old]
	‘*It was a bit weird [not giving hugs to friends], because you say hello, and when you say bye, it's normal to hug them, so it's a bit weird to be giving each other air hugs*.’ [Participant 5, female, 14 years]
	‘*As the rules have died a bit, I met with a friend and we just went on a run, socially distanced of course. It was nice to see them again in real life*.’ [Participant 20, male, 16 years old]
	‘*At some points I don't think we did social distance, like going to the shops and that*.’ [Participant 16, male, 15 years old]
	‘*Two of my friends have been round to each other's houses for barbeques, for tea, just to have a drink together but I mean they definitely don't stay 2 m away from each other. I know that for a fact ‘*cos *their garden's not big enough*.’ [Participant 8, male, 13 years old]
	‘*Well the fact is that none of them [protesters] are social distancing, they're all dotted together. I wouldn't be surprised if nearly, if not all of them, have got it*.’ [Participant 19, male, 15 years old]
	‘*I think morally in terms of the rules people should stay at home, but then again, this stuff that's happening, I totally get why people would protest*.’ [Participant 3, female, 12 years old]
Handwashing and hygiene	‘*I wash my hands more because obviously I don't want to spread the COVID-19 to other people or even affect myself because of my asthma. Handwashing is more of a scheduled thing now during lockdown or during this pandemic*.’ [Participant 7, male, 13 years old]
	‘*Everyone [at school] was washing their hands so much and I washed mine literally more than I've ever washed them, and they were all like dry and red and they've literally never been like that*.’ [Participant 21, female, 15 years old]
	‘*Oh, yes, he'll [younger brother] come into the house after a walk and go upstairs and I have to shout him down “did you wash your hands?*”’ [Participant 5, female, 14 years old]
	‘*It might help stop the spread of common ‘flu or colds in the future … I would imagine maybe less people are taking sick days off work if they're washing their hands more*.’ [Participant 14, male, 17 years old]
Self-isolation	‘*I've got older siblings and so I think if my parents and me stayed at home then they could do shopping and stuff like that for us. I think it would be quite easy to self-isolate*.’ [Participant 6, female, 13 years old]
	‘*If it's right now I won't care that much, I don't meet them [my friends] that much anyway. But if it's in the future, a month or two, I think I will care quite a lot and it will affect me quite a lot*.’ [Participant 16, male, 15 years old]
Enforcement of rules	‘*I've seen quite a lot on social media that everyone's meeting up and posting photos and stuff and that's showing a lot of people like “oh they're doing it, why can't I?” It's giving quite a bad message when people keep meeting up*.’ [Participant 2, female, 14 years old]
	‘*I remember when I first saw people proper close, if I could tell they weren't part of the same household, it annoyed me a lot. Why can't I do it then, if you can do it, it's not fair if you just decide to do it ‘cause I want to*.’ [Participant 20, male, 16 years old]
	‘*It's just selfish [that they are not social distancing] because they could be spreading the virus and that could potentially kill people*.’ [Participant 1, female, 12 years old]
	*‘It feels quite disheartening to see other people breaking them with very little concern for themselves or other people but there's not much I feel I can do really because people get quite defensive if you kind of say something to them*.’ [Participant 13, female, 15 years old]
	‘*The people I see bending the rules, they're young people like me, so it's understandable that you might be breaking the rules and being a bit rebellious essentially*.’ [Participant 14, male, 17 years old]
	‘*Teenage boys at least say whatever they like to other people, it doesn't usually change how they think about each other. You could tell someone off and you'd probably just be a social outcast*.’ [Participant 18, male, 13 years old]
	‘*My friend was walking to work and she had a hoodie and a rucksack on her back and an older man shouted at her because he thought she was going to meet friends … and she just said ‘I'm a key worker’ and she came into work and she was annoyed about it … I think he was quite aggressive*.’ [Participant 10, female, 16 years old]
	‘*There was one time when a runner – everyone was sort of queuing waiting their turn to go through [a path] - and the runner ran past everyone whilst screaming “2 m, 2 m”. Everyone just looked at him like what an earth are you doing? How can we keep 2 m when you run past panting? That was a bit weird. Everyone just kind of looked at each other like what was he thinking?*’ [Participant 14, male, 17 years old]

**Table 4:** Acceptability of vaccination against COVID-19.

	‘*When you're in a position when it [COVID-19] doesn't affect you, it would be selfish not to think of other people who you might pass it on to who it would affect*.’ [Participant 5, female, 14 years old]
	‘*I don't feel me or my brother are at risk, but obviously my grandparents are. I will try and protect us.*’ [Participant 16, male, 15 years old]
	‘*So we can just eradicate coronavirus. If everyone has it then we don't have to worry about it anymore*.’ [Participant 9, male, 13 years old]
	‘*I get the vaccine and then I could go back to school probably. That's the main thing that's different to routine at the moment*.’ [Participant 11, female, 17 years old]
	‘*When I was born there was some sort of vaccine which was giving people some sort of disease or something but was stopping another so I wouldn't want to get ones like that. But if one proved to be successful I'd happily have it*.’ [Participant 8, male, 13 years old]
	‘*I think it probably is better for them [older people] to get it just so they know that they're less in danger and they have more things to do because obviously the lockdown can't last forever and they're eventually gonna get older and older so they don't want to spend their last days staying in their house for 10 or 11 weeks*.’ [Participant 8, male, 13 years old]
	‘*Some people who don't believe that coronavirus even exists and it's completely made by the government. There's something that will just never change someone's mind, no matter how worldwide they are*.’ [Participant 13, female, 15 years old]

### 3.1 Impact of the COVID-19 pandemic on young people

None of the participants believed that anyone in their household had experienced COVID-19, although some young people were aware of cases affecting more distant members within their social networks. Therefore, the impact of the COVID-19 pandemic was usually felt through disruption to their schooling education, their social networks, and hobbies and extracurricular activities.

#### 3.1.1 Schooling and education

Since school closures, all of the young people interviewed were provided with home learning from their school. This usually involved being set work by the teachers which was uploaded by the young person via an online portal when completed. The learning undertaken at home was largely self-guided by the young people, with little or no additional input from teachers, even if requested. There was variation in relation to the acceptability of home learning, with the majority of young people reporting both advantages and disadvantages over face-to-face teaching in a classroom. There was no obvious difference in experience by age of the participant. Some young people preferred being able to independently self-direct their learning. However, disadvantages reported by young people included that they felt the home environment was less conducive to learning than the classroom environment (Table 2).

Some young people experienced difficulties in sustaining motivation and were more distracted at home, which led to worries about falling behind in their work. The young people not enrolled to sit national exams in summer 2020 reported little impact from not having to sit routine end of year tests. Mixed feelings were evident among those young people who were scheduled to sit national exams. Frustration over wasted effort towards their schooling and revision was evident, but this was counter-balanced by a reduction in stress from not having to prepare for and sit exams. There was also recognition that assessment based on teachers’ predicted grades could unfairly disadvantage young people who perform better in exams or do not have a good relationship with their teacher (Table 2).

At the time of data collection, most young people were unaware of plans to return to school, although some were attending school for short periods before the Summer Term ended. There was recognition that when schools fully re-opened there may be changes to how the school day would operate to adhere to social distancing guidance. Young people trusted the school to make changes to keep them safe and were not overly concerned about being at greater risk of contracting COVID-19 when they returned. The majority of young people were looking forward to their return to school and being able to spend time with their school friends. However, for some young people, school closures appeared to be more beneficial (Table 2).

#### 3.1.2 Disruption to social networks

Many young people reported limited face-to-face interaction with people outside their household in recent months. Most young people reported keeping in contact with friends through different digital platforms. A few of the participants appeared more isolated, reporting few interactions with people outside their household. As the guidelines around meeting up with members external to one's household relaxed in June 2020, older participants reported enjoying being able to spend time with friends. This was less frequently reported by younger participants who may have less independence than older participants and be more heavily reliant on parents to organise social events (Table 2).

#### 3.1.3 Hobbies and extracurricular activities

During lockdown, participants of this study often reported they had more time to pursue existing or new hobbies and extracurricular activities. Although some activities were unable to take place, some providers had switched to an online model of delivery to continue engaging with young people. Young people reported being more physically active than they would have been usually, going out for walks or runs with members of their household. There was improved confidence among some young people to cycle on roads when there was less traffic (Table 2).

### 3.2 Implementation of COVID-19 government guidelines

All study participants were aware of COVID-19 related government guidelines in relation to social distancing and increased handwashing and hygiene. Most young people also mentioned the requirement to stay at home and self-isolate in the event of being diagnosed with COVID-19. Reflecting the governmental guidance at the time, wearing face coverings was rarely considered by young people.

#### 3.2.1 Social distancing

Social distancing was most widely discussed among young people, reporting very high levels of household adherence to guidelines. Most young people reported very strict adherence to guidelines by not making physical contact, maintaining a two metre distance, and meeting friends or family members outside rather than inside. A few young people admitted they were not always strictly compliant, but these were usually minor infractions (Table 3).

Almost all young people perceived that others, mostly those of a similar age to themselves, may not adhere as strictly to socially distancing rules. At the time of interviews, in response to the death of an African American citizen in police custody, Black Lives Matter protests were taking place globally. Around half of the participants spontaneously discussed this in relation to whether social distancing could be maintained and the likely impact this could have on the ongoing pandemic. Young people had mixed feelings about whether people should have congregated at Black Lives Matter events (Table 3).

#### 3.2.2 Handwashing and hygiene

Family norms around hygiene measures appear to have changed, with young people reporting increased frequency and adherence to handwashing. Some young people reported having dry, cracked hands as a result of this change. Older family members may remind younger family members about adherence. Some young people recognised wider societal implications of increased handwashing and hygiene practices in relation to other infectious diseases (Table 3).

#### 3.2.3 Self-isolation

The need for self-isolation to contain COVID-19 was discussed by most young people during the interviews. None of the participants had to do this themselves, but appeared willing to if required. With the rules around social distancing becoming more permissive, some young people recognised greater difficulties in adherence to self-isolation if this resulted in missing out on social opportunities (Table 3).

#### 3.2.4 Enforcement of rules

Perceptions of non-compliance by other people invoked differing levels of social judgement by the study participants. This was mostly discussed in relation to social distancing, which was visible both in public settings and through social media. Many young people, both in the older and younger age groups, felt negatively towards people who were perceived to be ‘rule breaking’, explaining their actions were unjust, risky and selfish towards others. These young people felt largely powerless, or not confident enough, to communicate this directly to the perpetrators. Other young people were less affected by the actions of others, or felt it was not their place to enforce compliance. This may also be difficult among their peer groups. Where young people reported experiences of civic enforcement of ‘rule breakers’, these appeared to be viewed negatively (Table 3).

##### 3.2.4.1 Acceptability of vaccination against COVID-19

All young people interviewed were willing to be vaccinated against COVID-19 if a vaccine became available. Most were motivated by an altruistic desire to protect others at greater risk from COVID-19 than themselves. Others cited the importance of reducing the impact of the infectious disease among the population. Some young people recognised that the availability of the vaccine could help a return ‘back to normal’ (Table 4).

Others suggested they may not agree to be vaccinated if there was a potential for harm, for example through serious side-effects, or weak evidence for effectiveness. Some young people felt that the vaccine should be prioritised for population groups at greater risk. The young people interviewed were all pro-vaccine, but felt people with negative views about vaccines in general were unlikely to change their mind towards being vaccinated against COVID-19 (Table 4).

## 4 Discussion

The greatest impacts of the COVID-19 pandemic on young people have been through the indirect consequences of public health measures including school closures and social distancing guidelines, rather than from the disease itself. Young people reported adherence to public health guidelines which could be coupled with a sense of social injustice invoked where others were perceived to be non-compliant. There did not appear to be marked differences in levels of compliance by the age of participants. However, greater levels of freedom to meet and socialise with groups outside of their household were apparent among the older participants, which could contribute to perceptions of lower levels of adherence. This has the potential to influence ongoing adherence to public health guidance by young people, as has been shown in a recent study with an adult population [11].

To reduce the spread of COVID-19, school closures have been implemented in the majority of countries with significant implications for young people's education [12]. In general, young people in this study had continued their school work at home and appeared confident in their abilities to self-direct some of their learning. However, some anxieties were evident in relation to their perceptions of the effectiveness of home learning especially in preparation for external examinations. Data collection for this study largely took place using digital platforms, where young people used personal electronic equipment in their own bedroom or in a shared communal space without being interrupted by other members of their household. Reflective of their reported experiences, their home environment appeared to be conducive to home learning. The experiences reported in this study may not therefore be representative of the experiences of young people in other households throughout England or other households more widely where access to digital equipment, internet, or personal space is compromised.

In January 2021, schools in England were closed which is causing young people significant disruption to their education. The Chief Medical Officer has recently stated that young people are more likely to be harmed by the school closures, than from physical health problems resulting from COVID-19 [13]. Increased symptoms of mental distress have also been reported [5,6], in addition to increased loneliness and isolation, a lack of safe space, challenging family relationships and increased social media pressure [14].

In contrast, a recent survey undertaken in the south-west of England showed some young people have had reduced levels of anxiety during school closures. The authors attributed this to the removal of stress factors such as pressure of academic work and challenging peer relationships [15]. The qualitative findings of this study undertaken with young people who appeared to be from relatively affluent backgrounds lend support to there being perceived benefits from lockdown. Many participants of this study had additional time to pursue hobbies and extracurricular activities. In some cases, young people perceived their learning to be enhanced at home. For other young people who did not enjoy school, not having to attend school at designated times, or at all, could be of clear benefit. The reference to not having ‘to get up in the morning’ may also be relevant as research suggests teenagers have a natural tendency to fall asleep later and wake up later [16].

All participants of this study were favourable towards being vaccinated against COVID-19, primarily to protect higher-risk groups. A previous study examining young people's motivations to receive the HPV vaccine focussed on the benefits of protecting themselves from developing cancer [17]. It may be that greater attention should be given to young peoples' potential for altruism in a range of public health initiatives. Based on assumptions from influenza outbreaks, delivery of a COVID-19 vaccination programme to children and young people has the potential to be an effective strategy to interrupt transmission to older people [18] and reduce the requirement for additional school closures.

Controlling outbreaks in education settings is currently highly relevant to policy makers given the rising prevalence of COVID-19 infections among younger age groups. Schools-based models for delivery of adolescent vaccination programmes are widely acceptable among professionals, young people and their families [19] and can achieve high levels of uptake [[20], [21], [22]]. Future research could establish how best to develop a communication strategy for a future COVID-19 vaccine which aligns with the high levels of altruism reported by these young people.

### 4.1 Strengths and limitations

This study offers valuable insights into young people's views and experiences of the restrictions imposed upon them as a result of the COVID-19 pandemic. The sample was balanced by gender and included younger and older adolescents, but information relating to the socioeconomic status or ethnicity of the participants were not taken. Therefore, the findings from this study may not be representative of young people belonging to BAME groups or more deprived communities. The study used a rapid qualitative design in order to enable findings to be delivered in a timely way so they can be used to inform decision-making processes and policy. Presentation of findings took a broad approach, rather than focussing in-depth on a key issue.

## 5 Conclusions

Young people have experienced significant disruption to their education and social networks. High levels of compliance to government public health guidelines were reported suggested that young people are willing to play their part in reducing transmission of COVID-19. High levels of acceptability to be vaccinated were apparent.

## Funding

This work was supported by the Elizabeth Blackwell Institute, 10.13039/501100000883University of Bristol, the 10.13039/100010269Wellcome Trust ISSF3 grant 204813/Z/16/Z and QR 10.13039/100010764SPF (Quality-Related Strategic Priorities Fund), 10.13039/100014013UKRI 10.13039/501100013589Research England.

## Declaration of competing interest

There are no conflicts of interest declared.
